# Fire dragon cupping in treating a parturient woman with gastrointestinal dysfunction after cesarean section: A case report

**DOI:** 10.1097/MD.0000000000040201

**Published:** 2024-11-08

**Authors:** Huiying Zhang, Chunyan Ye, Xuefen Ye, Junhong Cai

**Affiliations:** a Obstetrical Department, Shenzhen Baoan Women’s and Children’s Hospital, Shenzhen, Guangdong, China; b Nursing Department, Shenzhen Baoan Women’s and Children’s Hospital, Shenzhen, Guangdong, China.

**Keywords:** case report, cesarean section, fire dragon cupping, postoperative gastrointestinal dysfunction

## Abstract

**Rationale:**

The postoperative gastrointestinal dysfunction after cesarean section is commonly presented as uncomfortable symptoms such as abdominal pain and distension. As a novel traditional Chinese medicine characteristic therapy, the fire dragon cupping can effectively enhance intestinal peristalsis and improve the gastrointestinal discomforts by using the manipulation and moxibustion heat to stimulate acupuncture points. The purpose of this report is to provide a new approach and new ideas for rapid rehabilitation of gastrointestinal dysfunction after cesarean section.

**Patient concerns:**

A parturient woman, 39 years old, pregnancy 4, delivery 2, underwent lower uterine segment cesarean section under intrathecal anesthesia due to scarred uterus. On the 3rd day after cesarean section, the anus still did not exhaust and defecate, so this parturient woman complained of obvious abdominal pain, abdominal distension, nausea, and vomiting.

**Diagnoses:**

The measured abdominal circumference was 96 cm, and the bowel sounds weakened on auscultation; the plain abdominal radiograph indicated a small amount of pneumomediastinum, thus the incomplete intestinal obstruction was considered.

**Interventions:**

The parturient woman was treated with fire dragon cupping treatment in her back and abdomen once a day, each time about 30 to 40 minutes, 3 consecutive days of treatment.

**Outcomes:**

On the 4th day after cesarean section, the parturient woman naturally had anal exhaust and defecated watery stools twice, without complaint of abdominal pain distension.

**Lessons:**

Under the guidance of the concept of rapid rehabilitation nursing in obstetrics, according to the principle of making gradual and orderly progress, risk management of gastrointestinal dysfunction after cesarean section is carried out in the early stage, the fire dragon cupping and individualized rehabilitation scheme are implemented, and the parturient women with gastrointestinal dysfunction after cesarean section are actively treated.

## 1. Introduction

At present, the cesarean section is mostly the cesarean section of lower uterine segment, which is the most common abdominal surgery and the most important obstetric surgery, and is an effective means to deal with high-risk pregnancies and abnormal deliveries, thus saving the save maternal and perinatal lives.^[1]^ Currently, the cesarean section rate in Europe and the United States is around 20%, and the cesarean section rate in China has increased again to 36.7% since the full liberalization of the second-child policy in China in 2016, and the cesarean section rate during the past 10 years has been higher than the threshold of 15% for cesarean section proposed by the World Health Organization.^[2–4]^

Under normal circumstances, the intestinal motility recovers within 24 hours, the gastric motility recovers within 24 to 48 hours, and the colon motility recovers within 48 to 72 hours after surgery. The postoperative gastrointestinal dysfunction (PGID) is a group of syndromes of nonmechanical and paralytic intestinal obstruction resulting from the failure of gastrointestinal function to recover on schedule due to the factors such as surgical stimulation, anesthetic drugs, pain, blood accumulation, and inflammatory response, which is clinically manifested by varying degrees of weakened intestinal peristalsis, weakened or absent intestinal sounds, abdominal pain, abdominal distension, nausea, vomiting, and cessation of anal exhaust or defecation,^[5]^ leading to extreme discomfort and anxiety in the parturient women, which is more prevalent in the clinic.

Therefore, we have applied a novel traditional Chinese medicine (TCM) characteristic therapy “fire dragon cupping” to improve the symptoms of gastrointestinal dysfunction, which is simple and easy for operation, with both comfort and clinical efficacy. This innovative therapy uses manipulation and moxibustion heat to stimulate acupoints, promote qi to activate blood, warm meridians to dissipate cold, strengthen the body resistance and supplement the deficiency, and regulate yin and yang, effectively remove the intestinal stagnation, promote intestinal clearance, enhance intestinal peristalsis, alleviate PGID, and thus accelerate the postoperative recovery process. The comfort and clinical efficacy of fire dragon cupping has been unanimously recognized by the parturient women.^[6]^

Here, we report the efficacy of fire dragon cupping in the treatment of a parturient woman with gastrointestinal dysfunction after cesarean section. The treatment procedure followed ethical principles and was approved by the institutional review committee in our hospital (2022072220384517).

## 2. Case report

A parturient woman, 39 years old, pregnancy 4, delivery 2, underwent lower uterine segment cesarean section under intrathecal anesthesia due to scarred uterus. She denied any history of medical, family, and psycho-social history. After the operation, the parturient woman had stable vital signs and were admitted to the ward and treated with sandbag compression on the abdomen and continuous electrocardiographic monitoring for 6 hours. Since the 1st day after cesarean section, the parturient woman complained of mild abdominal distension, the abdomen was soft to the touch, the measured abdominal circumference was 91 cm, without nausea and vomiting, the abdominal percussion was tympanic, the bowel sounds weakened on auscultation; on the 2nd day after surgery, this parturient woman complained of abdominal distension and pain, without anal exhaust, and the measured abdominal circumference was 93 cm, and the bowel sounds weakened on auscultation; on the 3rd day after surgery, the anus still did not exhaust and defecate, so this parturient woman complained of obvious abdominal pain, abdominal distension, nausea, vomiting, and the measured abdominal circumference was 96 cm, and the bowel sounds weakened on auscultation; the plain abdominal radiograph indicated a small amount of pneumomediastinum, thus the incomplete intestinal obstruction was considered. The abdominal ultrasonography revealed intestinal tube dilation and effusion, and no obvious free effusion was observed in the abdominal cavity. The parturient woman was treated with fire dragon cupping, and then the abdominal distension was relieved compared with before, and the measured abdominal circumference was 93 cm, and the bowel sounds were gradually increased; on the 4th day after surgery, the parturient woman naturally had anal exhaust and defecated watery stools twice, without complaint of abdominal pain distension. The specific operation method for the fire dragon cupping treatment is as follows:

① fire dragon cupping on the back of the body: the parturient woman was placed in a lateral position to expose her waist and back, and attention was paid to protecting personal privacy and keeping the abdomen warm; the parturient woman was guided to breathe deeply so that she could remain relaxed, the massage oil was used, it was checked whether the mouth of the fire dragon cup was intact and smooth, a specially made moxa cone was inserted into the fire dragon cup and then ignited with a lighter. It was observed that the moxa cone was burnt uniformly, then the operator measured the temperature of the mouth of the fire dragon cup using his or her hand. The fire dragon cupping was performed at the site of life-gate, and the rotational manipulation was mainly conducted. The gentle rolling and kneading could contribute to moxibustion heat penetration, it would be appropriate for the parturient woman to feel slightly hot without pain. 2 to 3 minutes later, the heat penetration was achieved; the fire dragon cup was rotated and moved up and down, back and forth, forward and backward along the bilateral bladder meridian (Weishu, Pishu, Ganshu, Danshu, Xiaochangshu, Dachangshu, Shenshu, and Pangguangshu) for 5 to 10 times, and finally the cupping treatment was enhanced at baliao acupoint (entwisting, kneading, pointing, and vibrating) for 3 to 5 minutes. The whole cupping treatment on the back of the body lasted for about 15 to 20 minutes.
② Fire dragon cupping on the abdomen of the body: the parturient woman was assisted to take a supine position and make her comfortable. Shenque was taken as the main acupoint, the operator measured the temperature of the mouth of the fire dragon cup using his or her hand, the fire dragon cup was placed firstly at the site of Shenque, the cup was gently moved when touching the abdomen at the beginning. After the parturient woman was adapted to the intensity of manipulation, the intensity of manipulation was enhanced appropriately, and the cupping was performed gently until the skin flushed. The back-shu points and front-mu points combination method was used, the cupping was performed successively on the acupoints such as Zhongwan, Guanyuan, Tianshu, Zhangmen, Shimen, Qimen, and Riyue, meanwhile, the plum flower petal-shaped mouth of fire dragon cup was used to stimulate the front-mu points by lightly pressing, pointing and squeezing. According to the direction of cesarean section incision, the gentle cupping was continuously performed in the parturient woman with an abdominal transverse incision at the acupoints such as Qihai and Zigong, and the suspended moxibustion was conducted mainly in the parturient woman with an abdominal vertical incision at the acupuncture points, and finally the cupping was performed along clockwise direction according to the colon status for 3 to 5 times, the whole cupping treatment on the abdomen of the body lasted for about 15 to 20 minutes. In the course of the operation, attentions were paid to the control of cup temperature, the amount of moxibustion, and the duration and degree of heating, it would be appropriate for the parturient woman to feel a sense of warmth without burning pain in local area; after the end of the treatment, the parturient woman was advised to pay attention to keeping warm and avoiding cold, she could drink a cup of light salt water if she was having symptoms such as dry mouth and tongue.

## 3. Discussion

TCM believes that the spleen and stomach are the source of Qi and Xue, the 6 Fu-organs transport and transform food and do not store them,^[7]^ it can be seen that the body fluid, and the essence of grain and water are dependent on the transportation and transformation of the spleen and stomach, the qi movement in spleen and stomach is closely related to gastrointestinal function, the trauma during cesarean section caused damage to the skin, tendons and muscles, resulting in deficiency of qi and blood, volume depletion of body fluid, obstruction of Fu-qi, and stagnation of meridians and collaterals, which are followed by abdominal pain and distension, and cessation of defecation and exhaust, leading to extreme discomfort and anxiety in the parturient woman. Therefore, it is urgently needed to explore a more comfortable and effective treatment scheme to promote the rapid recovery of gastrointestinal function after cesarean section.

The fire dragon cup is made by the firing of a mixture of black stone and purple sand; the cup mouth with a petal-shaped design is plated with gold and silver; the moxa cone is made of a pure authentic herb Chinese mugwort. The fire dragon cupping sets 6 functions in 1, including massage, scraping, moxibustion, kneading, ironing, and acupoint stimulation. This approach combines 10 methods such as kneading, grinding, pushing, compressing, stimulating the acupoints, shaking, flashing, vibrating, ironing, and pressing with the electromagnetic wave and photoelectrochemical effect of near-infrared light radiation in moxa-moxibustion, and it is a holistic natural treatment in TCM.^[8]^At present, the fire dragon cupping is primarily used in the treatment of diseases in the respiratory, nervous and digestive systems, spinal bone injury, and gynecological diseases, and this method has achieved good clinical efficacy in relieving pain, improving sleep and alleviating constipation.^[9]^ Meanwhile, the study of Li Shuiying has further confirmed that the application of the fire dragon cupping in the treatment of abdominal distension after cesarean section can significantly shorten the time of symptom disappearance and improve the comfort of the parturient woman.^[10]^

In the fire dragon cupping treatment, 12 muscle regions, meridians and collaterals and skin are taken as the sites of action, the herbal cream or essential oils is applied to penetrate into the subcutaneous area and enter into the body blood circulation by combined application of moxibustion and appropriate manipulation, and can play multiple roles such as promoting qi to activate blood, warming meridians to dissipate cold, strengthening the body resistance and tonifying deficiency, and regulating yin and yang.^[11]^ Inside the cup, the moxa cone made of authentic medicinal herbs are ignited, it can exert infrared radiation and warming effects during burning, so as to warm middle jiao to dispel cold and harmonize the qi and blood.^[12]^ The active ingredients of moxa can effectively regulate the nerve function and produce the effect of dredging the meridians, balancing the function of internal organs and adjusting the yin and yang, and also have the effect of sterilization, which are of positive significance in enhancing maternal immune function.

This woman had obvious abdominal distension, no anal exhaust, and weakened bowel sounds on auscultation after cesarean section, so her PGID was treated using the back-shu points and front-mu points combination method: Zhongwan acupoint is a mu point of internal organs, which can regulate gastrointestinal peristalsis bidirectionally, strengthen the spleen and stomach, calm the adverse-rising energy and promote water metabolism; Daheng acupoint is a shu acupoint on the spleen meridian of foot-Taiyin, which can strengthen the spleen and regulate the qi, eliminate the stagnation and remove the dampness, and regulate the stomach and intestines; Tianshu acupoint is a mu point of the large intestine, which is good at regulating the intestines and regulating qi-flowing for activating stagnancy and is a key acupoint in the abdomen, which can regulate the stomach in middle jiao, and invigorate spleen and regulate qi. Guanyuan acupoint is s a mu point of small intestine, which is good at supplementing qi and moving water, as well as benefiting qi and consolidating foundation. Corresponding acupoints were stimulated using the moxibustion and massage, and combined multiple acupoint moxibustion was conducted in a random sequence to facilitate meridian dredging, meridian-qi activation, promote qi and blood running, strengthen the spleen and stomach, balance the yin and yang, improve the insomnia, and further enhancing the body’s ability to resist disease, effectively relieve abdominal distension, and speed up the process of postoperative rehabilitation. The fire dragon cupping treatment was carried out once a day. After 3 consecutive days of treatment, the parturient woman had relieved abdominal distension compared with before, and her bowel sounds were gradually increased. On the 4th day after the operation, the parturient woman had anal exhaust, and naturally defecated watery stools twice, and without complaining of abdominal pain and distension.

In the Outline of Strategic Plan for the Development of Traditional Chinese Medicine (2016–2030)^[13]^ and the Opinions of the CPC Central Committee and the State Council on Promoting the Inheritance, Innovation and Development of Traditional Chinese Medicine,^[14]^ the future development direction and priorities of TCM in China have been clarified, and systematic plans have been put forward to comprehensively revitalize TCM, accelerate the reform of the medical and health system, build a medical and health system with Chinese characteristics, and promote the construction of a healthy China, so as to promote the health of parturient women. Application of fire dragon cupping in the treatment of PGID can achieve twice the result with half the effort, it is easy to operate and has no adverse effects, and it has been highly praised by the majority of patients and medical staff because of its unique therapeutic advantages.^[15]^ Especially since the COVID-19 epidemic spreads around the globe, it is obvious to all that Chinese herb medicines and TCM therapy have played a positive role and exerted a therapeutic effect in fighting the epidemic, which has promoted the unprecedented development of TCM characteristic technologies.^[16]^

## 4. Limitation

The limitation of this report is that fire dragon cupping technology requires specialized training to operate and has not yet been fully promoted.

## 5. Conclusion

Therefore, under the guidance of the concept of rapid rehabilitation nursing in obstetrics, according to the principle of making gradual and orderly progress, risk management of gastrointestinal dysfunction after cesarean section is carried out in the early stage, the fire dragon cupping and individualized rehabilitation scheme are implemented, and the parturient women with gastrointestinal dysfunction after cesarean section are actively treated, which can help to accelerate the recovery of gastrointestinal function and improve the comfort in parturient women after cesarean section, and reduce the incidence of complications, shorten the length of hospital stay, and reduce the burden of the state and society.

## Acknowledgments

The authors would like to thank our patients and all investigators involved in the conduct of this study.

## Author contributions

**Conceptualization:** Junhong Cai.

**Methodology:** Chunyan Ye.

**Resources:** Xuefen Ye.

**Writing – original draft:** Huiying Zhang.

**Writing – review & editing:** Huiying Zhang, Junhong Cai.
